# Does the Quality of Life in Operated Patients with Adolescent Idiopathic Scoliosis correspond with the Radiographic Parameters?

**DOI:** 10.5704/MOJ.1507.010

**Published:** 2015-07

**Authors:** MA Hisam, NS Siti, NP Jou, S Ghaneshinee, AR Shaharuddin, B Azmi, KFMM Mohd

**Affiliations:** Department of Orthopaedics and Traumatology, Universiti Kebangsaan Malaysia Medical Centre, Cheras, Kuala Lumpur, Malaysia

**Keywords:** Quality of life, SRS-22, adolescent idiopathic scoliosis

## Abstract

Objectives: Traditionally, scoliosis surgery is aimed at attaining a fused, balanced and painless spine. With improvement in surgical and instrumentation techniques, spine surgeons strive to achieve higher degree of Cobb’s angle and rib hump correction with the idea of greater patient satisfaction. The aim of this study was to determine the patients’ satisfaction using SRS-22 questionnaire and their correlations with the radiographic changes.

Materials and methods: A prospective study was conducted in a tertiary referral cen re using the Scoliosis Research Society-22 (SRS-22) questionnaire during the patients’ annual follow up, betwee February to April 2014. Thirtyseven patients who met the inclusion criteria were enrolled. Results: The mean pre-operative Cobb’s angles were 57.8o ± 12.7o and mean post-operative angle of 20.0o ± 10.4o, resulting in average correction of 65.9 ± 14.4%. Mean preoperative rib hump was 61.1 ± 15.4 mm with mean postoperative rib hump of 15.8 ± 17.8 mm, resulting in average reduction of 77.7 ± 23.7%. Mean of post-operative total SRS score was 4.1 ± 0.5. Using Spearman rank correlation, the percentage of Cobb’s angle correction versus the SRS-22 score showed correlation of 0.17 (P=0.33) while the percentage of rib hump reduction versus SRS-22 score showed a correlation of 0.11 (P=0.53).

Conclusion: In this study, the average total SRS-22 score was 4.1 ± 0.5 (range, 3.1-4.9) post-operatively indicating very high satisfaction rate overall. Despite attempts at greater curve correction and rib hump reduction, there is no direct correlation between patient satisfaction and radiographic parameters.

## Introduction

Scoliosis is a descriptive term used to describe side to side bending of the spine. Adolescent idiopathic scoliosis (AIS) is characterized by lateral deviation of the spine acquired between 10 to 18 years of age and by far the most common type of scoliosis in the adolescent Age group Usually these deformities have no major functional consequences but are rather perceived as serious cosmetic problem by the patients or their parents^1^. In order to diagnose AIS all the other causes should be excluded beforehand^2^. The prevalence of AIS is approximately 2% to 2.5% using cut-off point of 10o Cobb’s angle or more. The ratio of girls to boys with curves of 6-10o is equal but increases to a ratio of 5.4 girls for every one boy with curves of 21o or more^3^.

Adolescent idiopathic scoliosis patient often presents with symptoms and signs, which include shoulder asymmetry (one shoulder higher than the other), waistline asymmetry or tilt, trunk shift (comparing the chest or torso to the pelvis), and limb length inequality. Although AIS is a painless deformity and the patients have no weakness or movement problems, the cosmetic effect of scoliosis should not be underestimated. Adolescents are often very concerned about this aspect especially female patients. The initial diagnosis was usually made on clinical examination and confirmed with a whole spine radiograph in posterior anterior (PA) and lateral (Lat.) plane. Magnitude of the curve was measured based on Cobb's angle on the PA film. Curves initially 30° or less tended not to progress whereas curves more than 30° usually progressed. Single thoracic curves between 50° and 75° were the most likely to progress, an average of 29.4° or about 0.73°/year (29.4°/40.5 years)^3^.

Treatment for AIS divided into three modalities: observation, bracing and surgery. The surgery can be done by posterior approach for all type of curves or anterior approach for selected curves^4^. The gold standard for surgical treatment of adolescent idiopathic scoliosis is posterior spinal fusion with segmental instrumentation^5^. There are five main goals for surgical treatment for adolescent idiopathic scoliosis: to stop progression by achieving a solid fusion, achieve permanent deformity correction, improve appearance and improved perceived functional outcomes of cosmetic appearance, physical and psychosocial health. It is also to reduce the development of low back pain, degenerative changes, functional impairment and cardiopulmonary compromise in adulthood^6^.

The objective of this study was to assess the quality of life in operated patients - using Scoliosis Research Society-22 (SRS-22) questionnaire and to determine the correlations between quality of life with radiographic changes and the degree of Cobb’s angle correction and percentage of rib hump reduction.

## Materials and Methods

A prospective study was carried out between February to April 2014 at the Scoliosis Clinic, Universiti Kebangsaan Malaysia Medical Centre (UKMMC) after obtaining ethical clearance. Between February and April 2014, we enrolled post- operative patients with Cobb’s angle between 40 to 90 degrees on initial diagnosis when they showed up for their annual -follow up. They must be English-speaking females and have completed a minimum of two years’ follow up. All the patients were operated by the same group of surgeons, using a standard technique and the same spinal implant system. Patients with Cobb angle more than 90 degrees - and with other cause of scoliosis were excluded. We excluded AIS with more than 90 degree- curves as the treatment approach for these group of patient in our centre also included spinal osteotomy in the form of asymmetric pedicle substraction osteotomy or a posterior vertebral column resection depending on the curve.

Forty-four patients who met the inclusion criteria were reviewed, seven were excluded due to inability to obtain their radiographic images from UKMMC Medweb database. Written consent was taken from patients for participation in the study. For patients who were below 18 years of age, written consent was obtained from their guardians. All the patients recruited in this study were evaluated using the SRS-22 questionnaire.

Radiographic data collected included pre-operative and postoperative Cobb’s angle and also the pre-operative and postoperative rib hump length measured using the Medweb software. Cobb‘s angles were measured in the PA view of standing x-ray. The exact same levels of vertebrae were used in pre-operative and post-operative radiographic images. Rib humps were measured in millimeter from the most prominent point of the hump till the posterior vertebral bodies using lateral view of standing x-ray^7^.

The SRS-22 is a questionnaire specified to determine the quality of life on scoliosis patients. The English version of questionnaire used has been well validated internationally.

This questionnaire comprised 22 questions, which were categorized into five domains: Function, Pain, Self-image, Mental health and Satisfaction with Management; and an SRS total score. Each domain was scored from 1 to 5, with 1 indicating the worst outcome and 5 the best outcome^8,9,10,11^. In addition -; the age of menarche was obtained from each of the respondents. Spearman rank correlation was used to determine the correlation between radiographic changes and the SRS score. The data was analyzed using SPSS version 22.0.

## Results

The mean age at surgery for 37 patients was 15.3 ± 1.5 years (range, 13-18) and the mean age at menarche was 13.1 ± 1.1 years (range, 10-16). The average Cobb’s angle was 57.8 ± 12.7 (40.2-89.2). The average Cobb’s angle correction was 65.9 ± 14.4% (37.0-92.8). The average rib hump reduction was 77.7 ± 23.7% (30.9-100.0). The radiographic parameters were summarized in Table I. The SRS-22 Domain and their mean scores were summarized in Table II. The average total SRS score was 4.1 ± 0.5 (range, 3.1-4.9) post-operatively indicating very high satisfaction rate overall. The patients were mostly highly satisfied post-operatively (mean score > 4) with all domains except for self-image where they were only moderately satisfied. The detailed scores of each domain were summarized in table III. Using Spearman rank correlation, the percentage of Cobb’s angle correction versus the SRS score showed correlation of 0.17 (P=0.33, little or no correlation) while the percentage of rib hump reduction versus SRS score showed a correlation of 0.11 (P=0.53, little or no correlation).

**Table I:** Summary of parameters of radiographic score

Pre-operative Cobb’s angle (o)	57.8 ± 12.7 (40.2-89.2)
Post-operative Cobb’s angle (o)	20.0 ± 10.4 (3.7-46.5)
Percentage of Cobb’s angle correction (%)	65.9 ± 14.4 (37.0-92.8)
Pre-operative rib hump (mm)	61.1 ± 15.4 (28.9-95.7)
Post-operative rib hump (mm)	15.8 ± 17.8 (0.0-56.2)
Percentage of rib hump reduction (%)	77.7 ± 23.7 (30.9-100.0)

**Table II:** Summary of Post-operative SRS-22 Domain Scores

Function	4.2 ± 0.6 (2.8-5.0)
Pain	4.4 ± 0.6 (2.4-5.0)
Self-image	3.9 ± 0.6 (2.4-5.0)
Mental Health	4.1 ± 0.7 (2.4-5.0)
Satisfaction with Management	4.4 ± 0.7 (2.5-5.0)
Total	4.1 ± 0.5 (3.1-4.9)

**Table III:** Break up of each SRS Domain and Score with corresponding number of patients and their percentage

Function		5 (13.5)	19 (51.4)	13 (35.1)
Pain	1(2.7)	3 (8.1)	12 (32.4)	21 (56.8)
Self Image	1 (2.7)	9 (24.3)	20 (54.1)	7 (18.9)
Mental health	1 (2.7)	4 (10.8)	15 (40.5)	17 (45.9)
Satisfaction with management		3 (8.1)	13 (35.1)	21 (56.8)

## Discussion

**Fig. 1:**
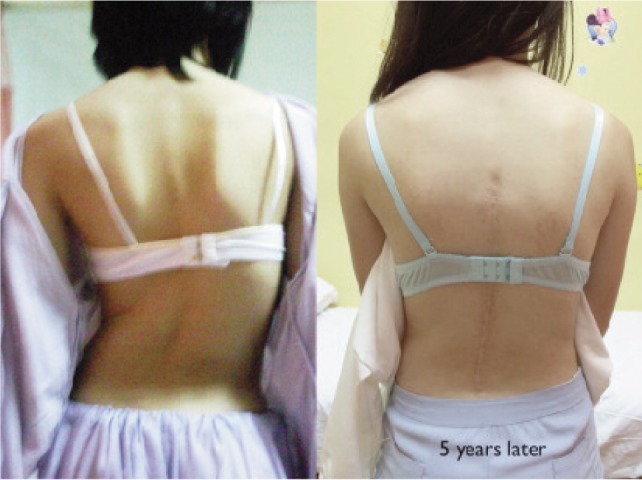
The cosmetic correction with surgery.

Traditionally, scoliosis surgery is aimed at attaining a fused, balanced and painless spine. The patient often presents with shoulder asymmetry, waistline asymmetry or tilt, trunk shift, and limb length inequality which and this cosmetic aspect of scoliosis should not be underestimated (Figure 1). Adolescents, especially female, are often very concerned about this aspect. With improvement in surgical and instrumentation techniques, spine surgeons strive to achieve a higher degree of Cobb’s angle and rib hump correction with the aim of achieving higher patient satisfaction especially from the cosmetic point of view (Figure 1).

In this study, the mean values of the SRS-22 domains were 3.9 for the Self Image domain, 4.1 for the Mental health domain, 4.2 for the Function domain, 4.4 for the Pain domain and Global satisfaction with Management domain. In general, the patients in this study were highly satisfied with their post-operative quality of life as indicated by the mean value of total SRS score of 4.1. The patients were mostly highly satisfied post-operatively (mean score > 4) with all domains except for Self- Image. However, there was no correlation between radiographic changes such as percentage of Cobb’s angle correction and percentage of rib hump reduction with the SRS-22 questionnaire scores.

Linda *et al* stated that there was little or no correlation between post-operative radiographic outcome of Cobb’s angle and questionnaire score. The multicenter study was conducted on 78 patients, less than 21 years old - using ‘The Scoliosis Research Society, Instrument of Outcome Assessment Scoliosis Patient Questionnaire’^7^. Ameri *et al* carried out a study on 40 adolescents with idiopathic scoliosis who were of age ten and above and showed that half of the total number of patients were satisfied with their back appearance post-operatively using a questionnaire that assessed physical and psychological measurements^12^. Besides Cobb’s angle, rib hump was also measured pre- and post-operatively to determine patients’ satisfaction. Theologis *et al* stated that there was a significant improvement in both lateral asymmetry and hump severity after spinal fusion. The researcher also had concluded that the size of rib hump measurement was not the only factor that affected the cosmetic appearance of the back^13^. Nevertheless, there was no correlation between percentage of rib hump reduction and patients’ satisfaction in our study. This study will provide assurance to the patients and also the guardians regarding the benefits of the surgery towards improving the quality of life post-operatively. Moreover, the surgeons should take the quality of life of the patient into consideration instead of focusing solely on improving the radiographic parameters.

## Conclusion

In this study, the average total SRS score was 4.1 ± 0.5 (range, 3.1-4.9) post-operatively indicating very high satisfaction rate overall. The patients were mostly highly satisfied post-operatively (mean score > 4) with all domains except for self-image where they were only satisfied. Among all the domains that were analyzed in SRS-22 questionnaire, Self-image was noted to have the lowest score. Despite attempts at greater curve correction and rib hump reduction, there was no direct correlation between patient satisfaction and radiographic parameters.
